# Prebiotic formation of cyclic dipeptides under potentially early Earth conditions

**DOI:** 10.1038/s41598-018-19335-9

**Published:** 2018-01-17

**Authors:** Jianxi Ying, Rongcan Lin, Pengxiang Xu, Yile Wu, Yan Liu, Yufen Zhao

**Affiliations:** 1 Department of Chemistry and Key Laboratory for Chemical Biology of Fujian Province, College of Chemistry and Chemical Engineering, Xiamen University, Xiamen, 361005 Fujian China; 2 Key Laboratory of Bioorganic Phosphorus Chemistry and Chemical Biology (Ministry of Education), Department of Chemistry, Tsinghua University, Beijing, 100084 China

## Abstract

Cyclic dipeptides, also known as 2,5-diketopiperazines (DKPs), represent the simplest peptides that were first completely characterized. DKPs can catalyze the chiral selection of reactions and are considered as peptide precursors. The origin of biochemical chirality and synthesis of peptides remains abstruse problem believed to be essential precondition to origin of life. Therefore, it is reasonable to believe that the DKPs could have played a key role in the origin of life. How the formation of the DKPs through the condensation of unprotected amino acids in simulated prebiotic conditions has been unclear. Herein, it was found that cyclo-Pro-Pro could be formed directly from unprotected proline in the aqueous solution of trimetaphosphate (P_3_m) under mild condition with the yield up to 97%. Other amino acids were found to form proline-containing DKPs under the same conditions in spite of lower yield. During the formation process of these DKPs, P_3_m promotes the formation of linear dipeptides in the first step of the mechanism. The above findings are helpful and significant for understanding the formation of DKPs in the process of chemical evolution of life.

## Introduction

As one of the simplest peptide derivatives in nature^1,2^, Cyclic dipeptides, also known as 2, 5-diketopiperazines (DKPs), which were ubiquitously observed in microorganism, plants and animals^3–10^, have been found to have many biological activities (e.g., antiviral, antibiotic, anticancer)^11–14^ and chiral catalysis properties^15^. Yamagata *et al*. and Imai *et al*. reported that the DKPs could act as activated intermediates in the peptides formation^16,17^. Moreover, the anti-microbial peptides could be formed by ring expansions of DKPs^18,19^. The origin of biochemical chirality and synthesis of peptides remains abstruse problem believed to be essential precondition to origin of life. Homochirality is an essential feature of life and is catalyzed by the enzymes which consist of mainly proteins. The process of catalyze chirality is extremely precise and complex that can not be covered at the beginning of life. There must be simpler forms to implement the function of the enzymes in the chemical evolution process. The catalytic systems being included range from small-molecule (e.g., nanoparticles, transition metals, organic molecules) to enzymes^20^. Something as simple as intrinsic reactivity may have driven the prebiotic evolution. DKPs, like (Ni, Fe)S as small-molecule catalysis alternative to enzyme proteins^21,22^, that exert the chiral catalytic function prior to appearance of the enzyme protein and may not themselves have a direct evolutionary relationship to the enzyme protein in the origin of life process on earth. Meanwhile, DKPs also are considered as peptide precursors^23^. Thus, it is reasonable to believe that the DKPs could have played a key role in the origin of life on earth. Generally, the DKPs were synthesized from protected amino acids in non-aqueous solutions with the presence of bases or acids^15,24^. Nonappa *et al*. and Jainta *et al*. also reported the synthesis of DKPs from free amino acids under microwave conditions^2,25^. To our knowledge, there are limited report involving the formation of DPKs under prebiotic conditions.

Trimetaphosphate (P_3_m), as a good water-soluble reagent, shows great ability in the polymerization of small biomolecules such as amino acids and nucleotides^26–28^. Since most of the phosphates are insoluble apatites, P_3_m may be essential for prebiotic chemical evolution process on the primitive earth. P_3_m is widespread in microorganisms^29^, and is also found from volcanic activities^30^. The dipeptides formation from alanine or glycine with the presence of P_3_m in aqueous solution has been widely known for several decades^26^. Here, we attempted to extend this reaction to the formation of other dipeptides. Unexpectedly, the cyclo-Pro-Pro was the major dipeptide product when proline was reacted with P_3_m and only trace of linear-Pro-Pro was detected. Due to the importance of DKPs in peptides chain elongation and chiral catalysis properties that may have been impact on the prebiotic evolution, it prompted us to explore whether proline-containing DKPs could be produced within the prebiotic chemical inventory.

## Results and Discussion

### The reaction of L-amino acids with P_3_m in aqueous solution

P_3_m was treated with ten different L-amino acids (Met, Phe, Gly, Ala, Val, Pro, Ser, Arg, His and Asp) in aqueous solution at pH 10.7 and 35 °C for 7 days, respectively. The pH value of the reaction mixture was adjusted with 10 M sodium hydroxide during the whole reaction process.

Subsequently, the above resulting solutions were analyzed by HPLC-MS. The results showed that six linear dipeptides were formed in the reactions with corresponding amino acids (Met, Phe, Gly, Ala, Val and Pro), while the remaining amino acids (Ser, Arg, His and Asp) did not form the corresponding dipeptides with the activation of P_3_m. (Table 1 and Supplementary Fig. S1).

**Table 1:** Reaction conditions and linear dipeptide detections results of amino acids reaction with P_3_m.

Amino acids	Initial reaction conditions			Linear dipeptides
	Initial pH	Temperatures (°C)	Time (days)	
Met	10.7	35	7	√ ^a^
Phe	10.7	35	7	√
Gly	10.7	35	7	√
Ala	10.7	35	7	√
Val	10.7	35	7	√
Pro	10.7	35	7	√
Ser	10.7	35	7	×^b^
Arg	10.7	35	7	×
Asp	10.7	35	7	×
His	10.7	35	7	×

^a√^The corresponding linear dipeptide was detected by HPLC-MS. ^b×^The corresponding linear dipeptide was not detected by HPLC-MS.

### The discovery of the formation of cyclo-Pro-Pro in aqueous solution

**Figure 1:**
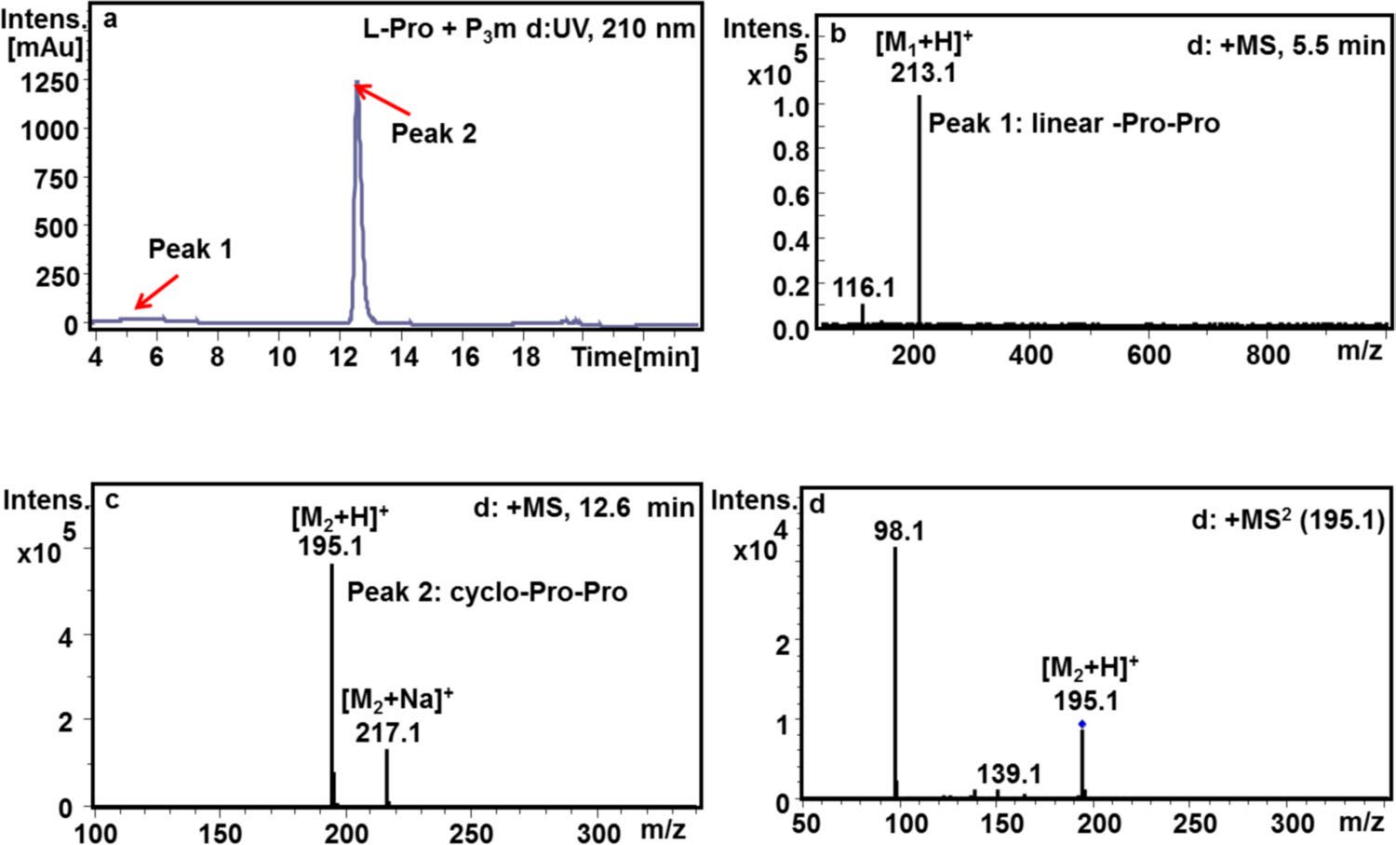
HPLC-MS profile for the reaction products of the L-Pro with P_3_m. (**a**) HPLC profile for the reaction products of the L-Pro with P_3_m at 210 nm. (**b**) MS profile for the LPD (peak 1, M_1_ = 212, R.T. = 5.5 min). (**c**) MS profile for the cyclo-Pro-Pro (peak 2, M_2_ = 194, R.T. = 12.6 min). (**d**) MS^2^ profile for the cyclo-Pro-Pro (M_2_ = 194, R.T. = 12.6 min).

From Fig. 1a, there were two absorption peaks (R. T. = 4~6.4 min, and 12.6 min) at 210 nm with the molecular ion peak ([M + H]^+^) at *m/z* 213 and *m/z* 195 (Fig. 1b,c), which correspond to linear- and cyclo-Pro-Pro, respectively. To our surprise, cyclo-Pro-Pro as the major product was also detected in the reaction mixture for proline and P_3_m at pH 10.7 after 7 days (Fig. 1). The relative abundance of linear-Pro-Pro (LPD) and cyclo-Pro-Pro was calculated by the integral area of 1.6% and 98.4%, respectively. Additionally, the fraction of the major peak 2 was collected and analyzed by NMR, which further confirms that the absorption peak at 12.6 min was cyclo-Pro-Pro (Supplementary Fig. S2). This shows when proline is treated with P_3_m in aqueous solution, not only the LPD but also predominantly the cyclo-Pro-Pro can be produced.

**Figure 2:**
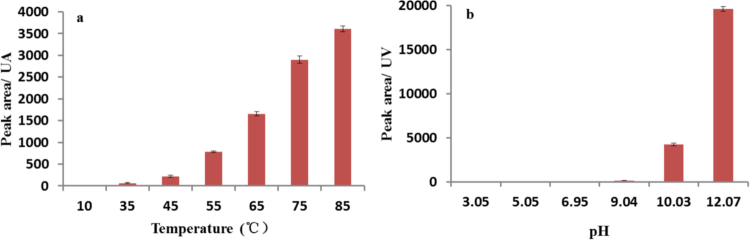
Effects of temperature and pH on the formation cyclo-Pro-Pro in the reaction of Pro with P_3_m. All reactions were controlled under standard conditions. (**a**) The formation of cyclo (Pro-Pro) at various temperature was assayed at pH 10.7. (**b**) The formation of cyclo-Pro-Pro at various pHs was assayed at 85 °C.

To further optimize the cyclization reaction conditions, L-Pro as the model amino acid was reacted with P_3_m in aqueous solution at different temperatures and pH value. When the reaction temperature raised to 85 °C, a higher cyclic dipeptide yield was obtained, by contrast when the reaction temperature decreased to 10 °C, no cyclic dipeptide was detected (Fig. 2a). Considering that the amino acids are easily racemic at high temperature and pH conditions, the optimum pH for this reaction is found to be around 10.7 at 55 °C (up to 97%, quantitation ^1^H-NMR), while under neutral or acidic reaction conditions, no cyclic dipeptide was obtained (Fig. 2b). (${[\alpha ]}_{{\rm{D}}}^{20}$ = −81.6 (CHCl_3_, c = 1.00 g/mL)) Similarly, the cyclo-D-Pro-D-Pro could also be obtained in 86.4% yield under the same condition when D-proline was treated with P_3_m (Supplementary Fig. S3).

Besides L- and D-Pro, the reactions of hydroxyproline, pyroglutamic acid and nipecotic acid with P_3_m under the same conditions were also studied. Only the hydroxyproline could form the cyclic dipeptide, which was assigned by ^1^H NMR according to the previous report^2^. (Supplementary Fig. S8) These results suggest that pyrrole-containing α-amino acids are favored when reacted with P_3_m to form the cyclic dipeptides under the potentially prebiotic conditions. The detailed studies about the formation features of the relative proline analogs are on-going in our lab.

### The cyclization reaction of proline with other amino acids (AAs)

It is well known that proline-containing DKPs are frequently found as an integral part of many natural products^31^. Therefore, we wondered if proline-containing DKPs can be formed from the reaction of L-proline with another amino acid with the presence of P_3_m under the potentially prebiotic conditions, namely aqueous solution, basic pH, and low temperature.

With the optimized reaction conditions for cyclo-Pro-Pro, we then investigated the reaction of P_3_m and Pro with glycine or alanine firstly. Unfortunately, no cyclo-Pro-Gly or cyclo-Pro-Ala was detected by HPLC-MS in the resulting mixture. We proposed that, under the above optimized reaction conditions, cyclo-Pro-Pro is the only preferred dipeptide to form, rather than the heterocyclic dipeptide.

**Figure 3:**
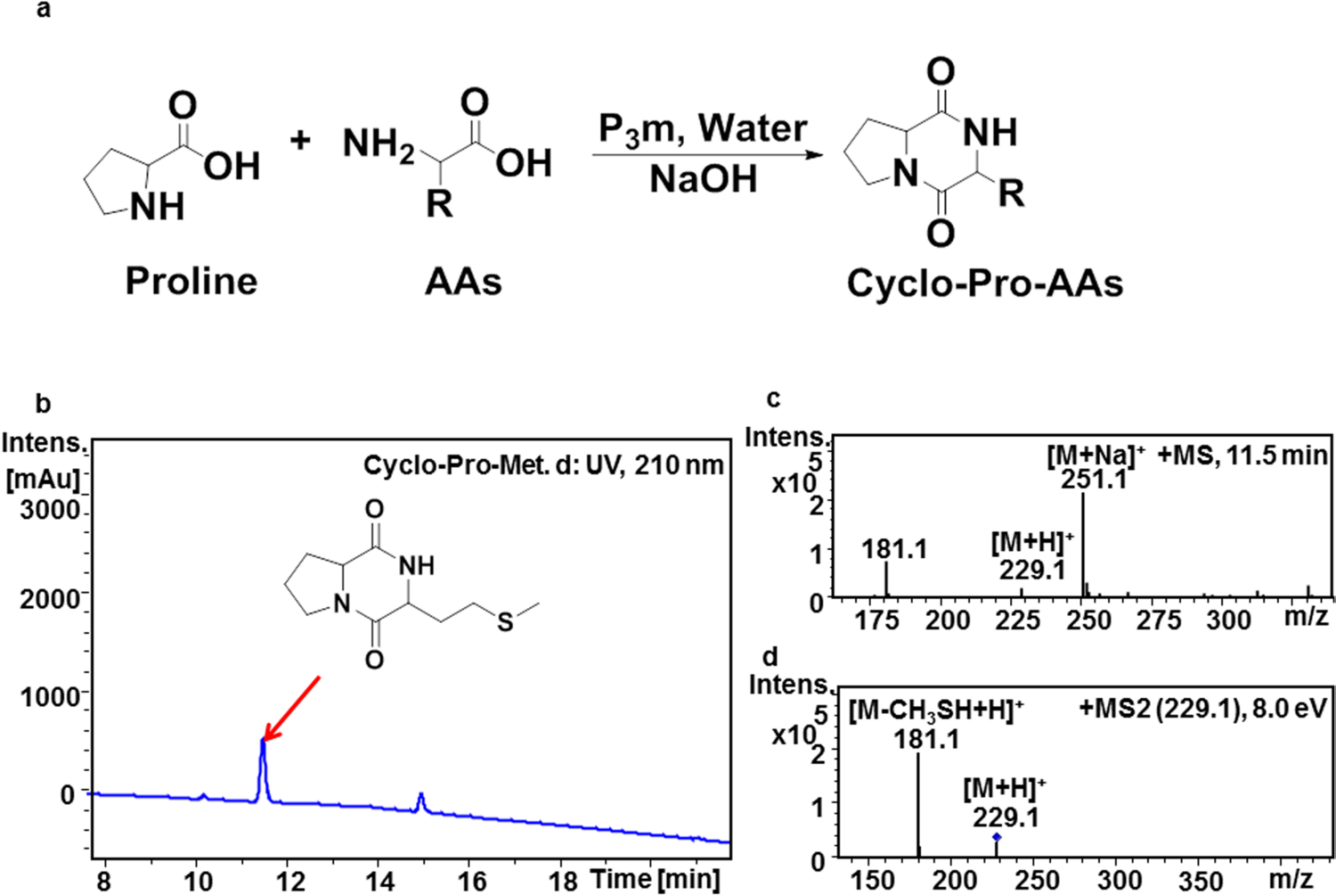
Reaction pathway and HPLC-MS/MS analysis of cyclo-Pro-AAs products. (**a**) The reaction pathway of cyclo-Pro-AAs, AAs stands for amino acids; (**b**) HPLC profile for the reaction product cyclo-Pro-Met of Pro with Met and P_3_m in aqueous solution; (**c**) MS profile for the product cyclo-Pro-Met (M = 228); (**d**) MS^2^ profile for the product cyclo-Pro-Met. HPLC-MS/MS profile for the reaction products of Pro with other amino acid could be found in the Supplementary Fig. S5.

**Figure 4:**
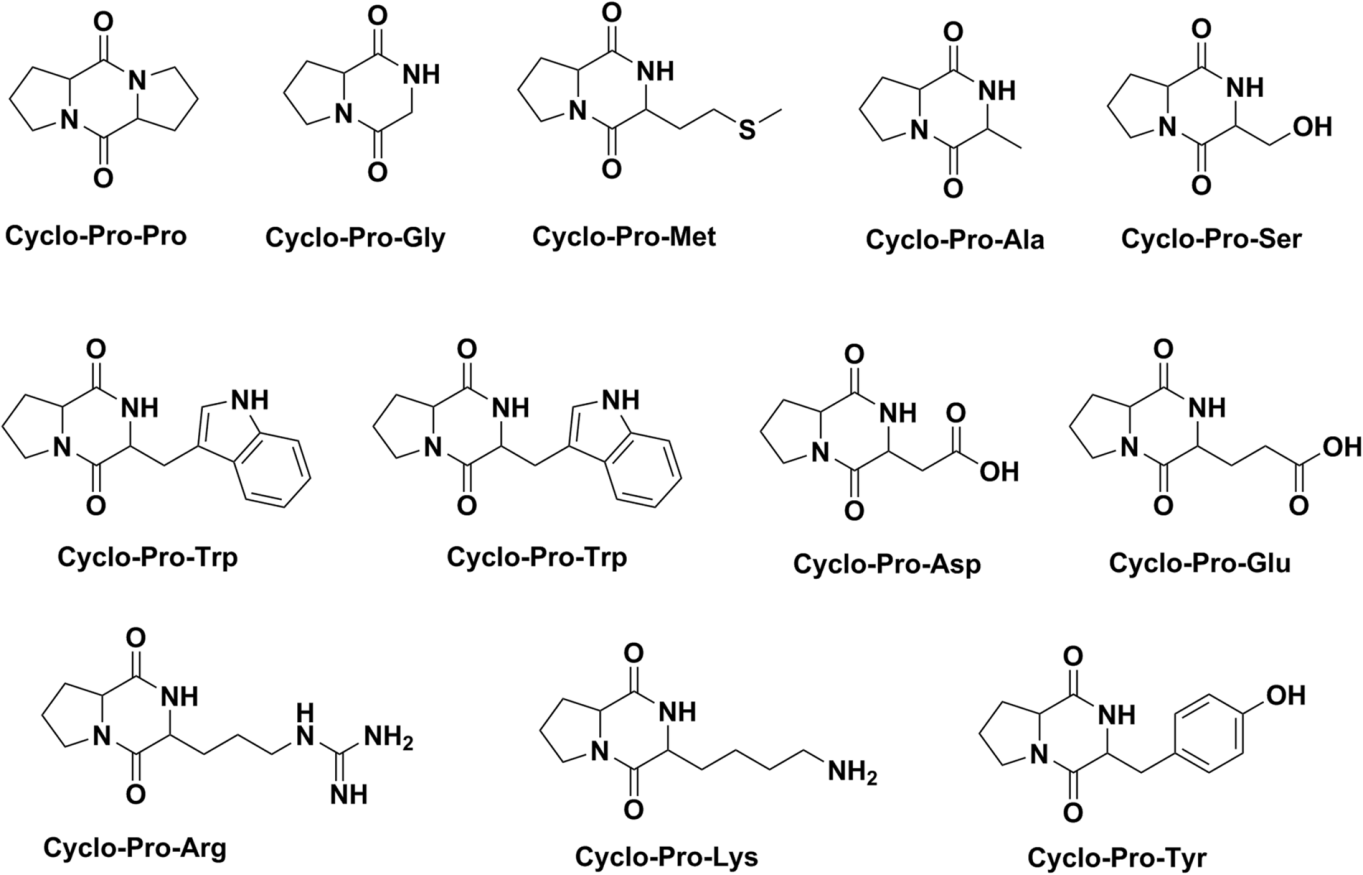
Representative structures of the product cyclo-Pro-AAs.

We then optimized the reaction conditions by reducing the reaction temperature to 35 °C to passivate the reaction activity of proline. Experiments of 12 natural amino acids (Ser, Ala, Glu, Gly, Arg, Asp, Lys, Phe, Met, Tyr, Trp, and His) were carried out in aqueous solution at an initial pH 10.7 at 35 °C for 7 days. The pH was not adjusted during the whole process. The proline-containing DKPs were all detected from all the resulting solutions by HPLC-MS except for the corresponding proline-containing DKP of cyclo-Pro-His. (Table 2, Fig. 3) All the corresponding structures of the products cyclo-Pro-AAs were summarized in Fig. 4.

**Table 2:** Reaction conditions and detection results of the products from the reaction of Pro with other amino acids and P_3_m.

P_3_m + Pro + AAs^a^	Initial reaction conditions			Relative abundance based on the total three proline-containing peptides^b^		
	Initial pH	Temp. (°C)	Time (days)	Cyclo-Pro-AAs	Linear-Pro-AAs	Cyclo-Pro-Pro
Ser	10.7	35	7	45.8%	×^c^	54.2%
Ala	10.7	35	7	32.5%	×	67.5%
Glu	10.7	35	7	18.4%	×	81.6%
Gly	10.7	35	7	16.5%	×	83.5%
Arg	10.7	35	7	15.4%	×	84.6%
Asp	10.7	35	7	12.8%	×	87.2%
Lys	10.7	35	7	6.2%	×	93.8%
Phe	10.7	35	7	2.2%	92.4%	5.4%
Met	10.7	35	7	1.9%	74.3%	23.8%
Tyr	10.7	35	7	1.7%	64.3%	34.0%
Trp	10.7	35	7	0.6%	80.7%	18.6%
His	10.7	35	7	×	×	×

^a^AAs: amino acids. ^b^The relative abundance were calculated by the integral area at 210 nm. As the Fig. 1 showed that linear-Pro-Pro was easy to form cyclo-Pro-Pro, so the linear-Pro-Pro was not included in the proline-containing products; ^c×^The corresponding product was not detected by HPLC-MS.

Following the progress of the reactions, the pH of the solution dropped, and when the pH was below 9, the reactions were almost stopped. To obtain the amount of the purified heterocyclic dipeptide, next experiments were performed for Met and Gly in the mixtures with Pro and P_3_m in aqueous solution at pH 10.7 and 35 °C for 7 days, respectively. The pH value of the reaction mixture was controlled using 10 M sodium hydroxide during the whole reaction process. Unfortunately, the predominant cyclic dipeptide products were the cyclo-Pro-Pro, whereas the heterocyclic dipeptide were obtained at very small amount. The related NMR spectra are shown in supporting information (Supplementary Figs S6 and S7).

It is worth noting that, although the yields of heterocyclic dipeptides are fairly low, even trace, these results are of significant meaning in the evolution process of life. The reactivity distinction of different amino acids may be caused by the follow reasons: 1) Proline prefers to form cyclo-Pro-Pro, and the main cyclic dipeptide product of these reactions is cyclo-Pro-Pro; 2) The amino acids with small steric hindrance side chains are more likely to form corresponding heterocyclic dipeptide, such as Ser, Ala and Gly. 3) Aromatic amino acids with big side chains are more inclined to form relevant linear dipeptides with proline, such as Phe and Trp. The possible reason is the steric effect of the aromatic amino acid side chains, which makes it harder for linear dipeptides to further cyclize into DKPs.

### The reaction mechanism of proline cyclization in aqueous solution

**Figure 5:**
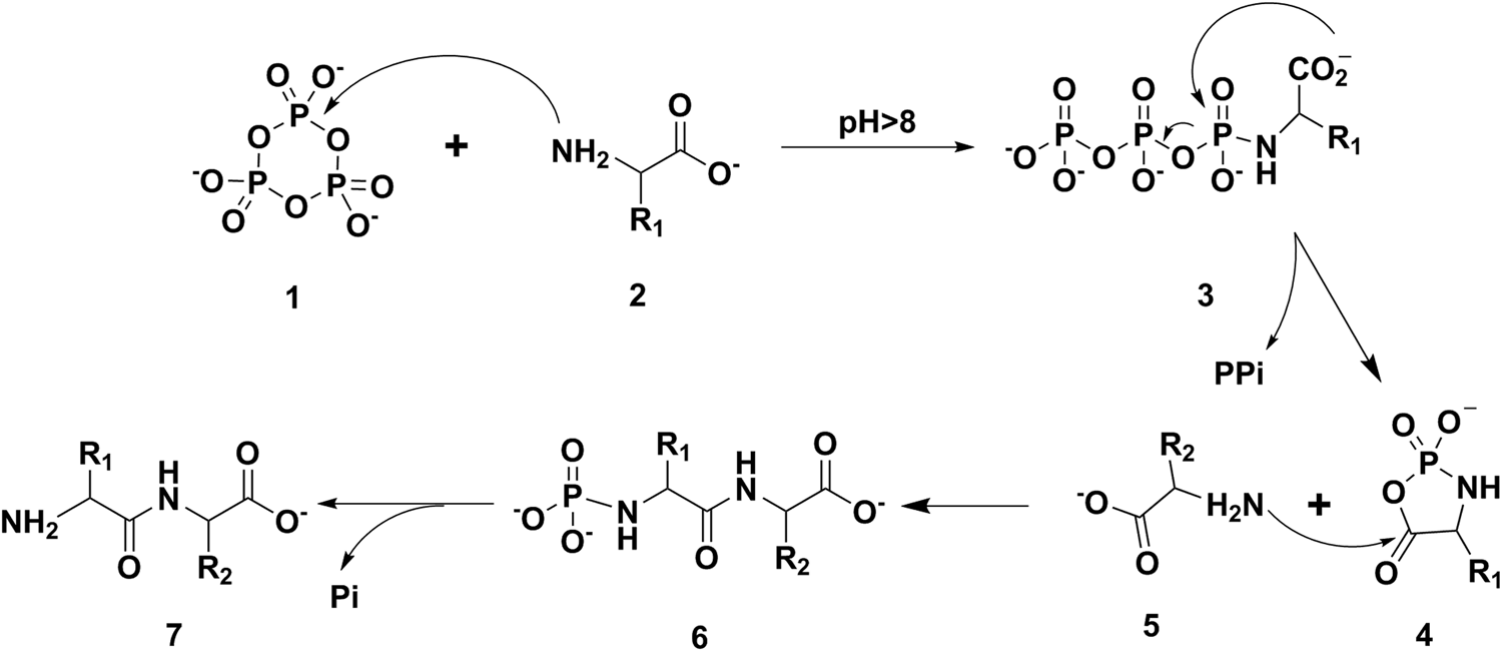
Mechanism for the linear dipeptide formation by P_3_m activation.

We then turned our attention to the mechanism of dipeptide cyclization. Given the *N*-phosphorylation of amino acids leading to linear dipeptide formation have been reported in the reaction of amino acids with P_3_m aqueous solution^32–34^, we speculate that the cyclization of dipeptide may undergo two steps, *N*-phosphorylation of amino acids and the cyclization of linear dipeptide, shown in Fig. 5. However, whether the cyclization of linear dipeptide is promoted by P_3_m is still unknown.

**Figure 6:**
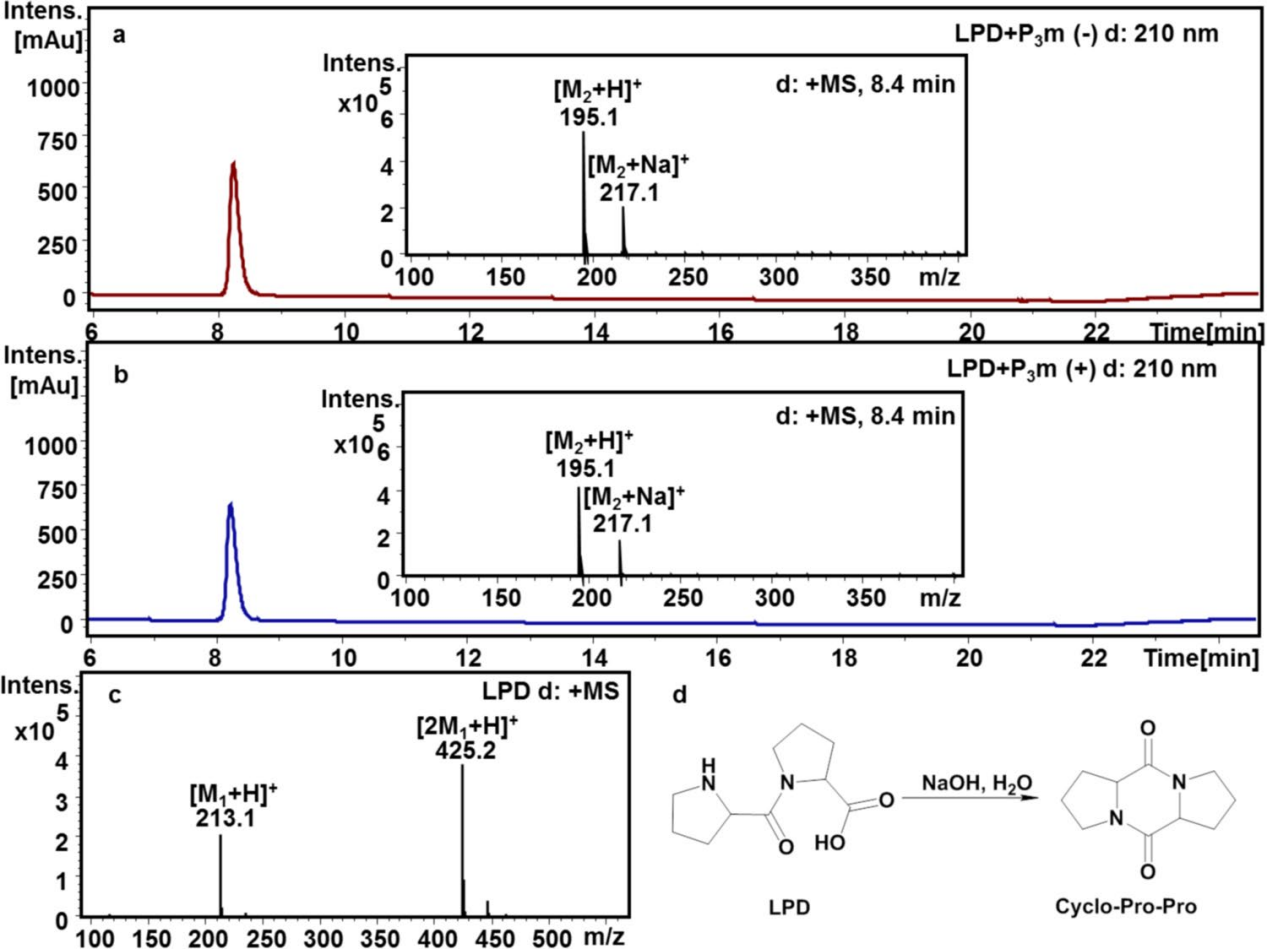
HPLC/MS profiles for the reaction products of LPD with or without P_3_m. (**a**) The HPLC/MS spectra of LPD in alkaline reaction solution without P_3_m. Integral area of the product cyclo-Pro-Pro is 6620 mAu·s (R.T. = 8.1~8.6 min, M_2_ = 194); (**b**) The reaction mixture of LPD in alkaline solution with P_3_m. Integral area of the product cyclo-Pro-Pro is 6929 mAu·s (R.T. = 8.1~8.6 min, M_2_ = 194); (**c**) The MS spectrum is derived from freshly prepared LPD aqueous solutions (M_1_ = 212); (**d**) A possible reaction mechanism of the cyclo-Pro-Pro formation through the cyclization of LPD in the alkaline aqueous solution.

In order to test this hypothesis, the LPD was chosen as a model compound in the following experiments. The solutions of LPD with or without the presence of P_3_m were incubated at pH 10–11 and 55 °C for 1 d. The resulting reaction mixtures were analyzed by HPLC-MS to determine the yield of cyclo-Pro-Pro. Interestingly, cyclo-Pro-Pro was detected with a similar yield in the two reactions (Fig. 6a,b). These results indicated that P_3_m was not involved in the cyclization process of LPD, and cyclo-Pro-Pro was generated through LPD cyclization in alkaline solution spontaneously.

**Figure 7:**
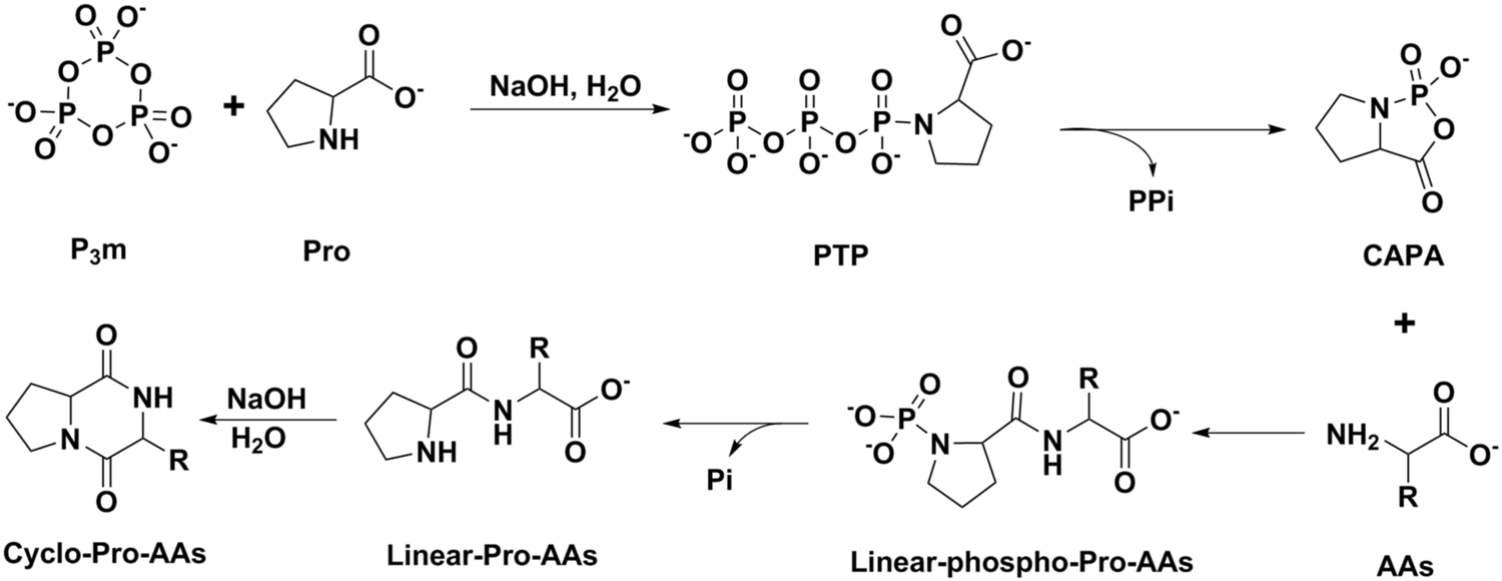
Mechanism for the formation of proline-containing cyclic dipeptides PTP: Proline triphosphate; CAPA: Cyclic acylphosphoramidates.

Comparing the results in Table 1 and Table 2, in which Ser, Arg and Asp on their own could not form corresponding linear dipeptides in aqueous solution of P_3_m, whereas their reaction with proline could form the corresponding proline-containing cyclic dipeptide (Cyclo-Pro-AAs) in aqueous solution of P_3_m. Based on the above observation and taken into account the linear dipeptide formation mechanism (Fig. 5) and the formation the cyclic proline dipeptide from linear proline dipeptide spontaneously (Fig. 6d), a possible mechanism for the formation of cyclo-Pro-AAs can be deduced as in Fig. 7. In general, the Pro first reacts with P_3_m to form proline triphosphate (PTP), then PTP is hydrolyzed to pyrophosphate (PPi) and cyclic acylphosphoramidates (CAPA); soon afterwards, the other amino acids (such as Ser, Arg and Asp) attack CAPA to form the phosphorylated linear proline-containing dipeptide (linear-phospho-Pro-AAs); and then the linear-phospho-Pro-AAs are hydrolyzed to inorganic phosphate (Pi) and linear-Pro-AAs; subsequently, the linear-Pro-AAs spontaneously form cyclo-Pro-AAs under alkaline conditions.

## Conclusions

It is well known that the formation of peptides in prebiotic environment is one of the most overlooked subjects in the study of chemical evolution of life^26,35,36^. The DKPs play an important role in the origin and development of early life^2,22,37^. In this work, we found that free amino acids, especially proline, could condense to afford DKPs under potentially prebiotic alkaline aqueous conditions with high yield. Although the yields of proline-containing hetero DKPs are low, these results are of significant meaning in the evolution of life process. These findings are very helpful to understand the formation of DKPs in the early chemical evolution process of life and provide some support for well-understanding the role of DKPs in the process of chemical origin. The reactivity described here provides a possibility of prebiotic chemical evolution reactions that occur without enzymes, but with a simpler small-molecule catalyst (DKPs).

## Methods

All the reactions were setup as follows: 0.3 mmol amino acids, 0.3 mmol sodium trimetaphosphate (P_3_m), 0.3 mmol proline (if used) were added in a glass vial or pasteur tube containing 3 mL deionized water, to which the pH of the reaction mixture was adjusted to 10~11 using 10 M sodium hydroxide. Reaction vials or tubes were tightly sealed and placed at 35 °C for 7 d or heated at 55 °C for 3 d.

Other methods and instruments details are provided in Supporting Information.

## Electronic supplementary material

Supplementary Information
