# Supplementary material for: Prebiotic formation of cyclic dipeptides under potentially early Earth conditions
Source: Sci Rep. 2018 Jan 17;8:936. doi: 10.1038/s41598-018-19335-9 (PMC5772641; doi:10.1038/s41598-018-19335-9)
Supplement: Supplementary file 1 — Supplementary Information [file 41598_2018_19335_MOESM1_ESM.pdf]

Prebiotic formation of cyclic dipeptides under potentially early Earth conditions

Jianxi Ying [1], Rongcan Lin [1], Pengxiang Xu [1], Yile Wu [1], Yan Liu [1, *] & Yufen Zhao [1, 2, *]

[1] Department of Chemistry and Key Laboratory for Chemical Biology of Fujian Province, College of Chemistry and Chemical Engineering, Xiamen University, Xiamen 361005, Fujian, China

[2] Key Laboratory of Bioorganic Phosphorus Chemistry and Chemical Biology (Ministry of Education), Department of Chemistry, Tsinghua University, Beijing 100084, China

Contents

1 Materials and Instruments........ 2

2 Homogeneous Amino acids mixed with P_3m reactions........ 3

3 NMR spectra of product cyclo-Pro-Pro........ 5

4 Spectra of product cyclo-D-Pro-D-Pro........ 6

5 Reaction products analysis of proline mixed with amino acids and P_3m........ 8

6 NMR spectra of product cyclo-Pro-Met........ 11

7 NMR spectra of product cyclo-Pro-Gly........ 12

8 The crude products' NMR spectrum of hydroxyproline mixture with P_3m........ 13

1 Materials and Instruments

Proline, methionine, glycine, valine, histidine, alanine, serine, phenylalanine, arginine, aspartic acid, glutamic acid, lysine were obtained from Aladdin Ltd.. Unless otherwise noted, these amino acids used here are L-configuration. Trimetaphosphate was purchased from Sigma Aldrich. Analytical reagents included methanol (HPLC grade), acetonitrile (HPLC grade) were purchased from SpectrumChemical. Formic acid and ammonium acetate were purchased from Sigma Aldrich. Ultrapure water (18.2 MΩ cm) from a Milli-Q water purification system (Millipore, Bedford, MA) was used to prepare solutions and the mobile phase.

The ^{1}H and ^{13}C NMR measurements were performed on Bruker 400 MHz, 600 MHz or 850 MHz NMR spectrometer at ambient temperature. The pure product was isolated and purified by liquid chromatography.

MS and MS^2 were performed on a Bruker micrOTOF-Q Ⅱ system in positive mode. The HPLC was performed on Agilent 1260 Infinity system and fitted with an Agilent TC- C18, 5 μm, 4.6 mm × 150 mm column or a Supelco LC-NH_2, 5 μm, 4.6 mm × 250 mm column. According to the different properties of dipeptides, one of the following two chromatographic procedures was used. a) For the linear dipeptides containing glycine and alanine, the solvent A was 5 mM ammonium acetate and solvent B was acetonitrile. The HPLC flow rate was 1 mL /min with LC-NH_2 column. A 22 min isocratic gradient method was used: 0-22 min, 70 % B. Column temperature was maintained at room temperature. b) For the others dipeptides, the solvent A was 0.1% formic acid and solvent B was methanol. The HPLC flow rate was 0.8 mL /min with TC-C18. A 29 min gradient method was used: 0-15 min, 5-30 % B; 15-20 min, 30-70 % B; 20-22 min, 70 %B, 22-24 min 70-5 %B, 24-29 min 5%. Column temperature was maintained at room temperature.

MS instrument parameters were as follows: Capillary voltage 4500 V, Nebulizer pressure 2 bar, Dry gas 8 L/min, Dry temperature 200 ℃. Mass spectra were registered in the scan range from *m/z*=50 to 1000. For ESI-MS, about 1/10 of the eluate from LC was introduced through a splitting T valve. To on-line detection the reaction product by HPLC-MS, we set up the divert valve of MS instrument as follows: 1) when divert valve in waste position, the valve can be used for switching HPLC flows directly to waste about 3 min; 2) after that, divert valve in source position, the valve can be used for switching HPLC flows directly to MS.

2 Homogeneous Amino acids mixed with P_3m reactions

0.3 mmol sodium trimetaphosphate (P_3m) was mixed with 0.3 mmol amino acids (Met, Phe, Gly, Ala, Val, Pro, Ser, Arg, His, Asp) in 3 mL water, respectively. The pH of the reaction mixture was adjusted to 10~11 using 10 M sodium hydroxide. Then the reaction mixtures were placed at 35 ℃ for 7 d. The reactions were quenched by adding 6 M HCl. The crude products were filtered and analyzed by HPLC-MS.

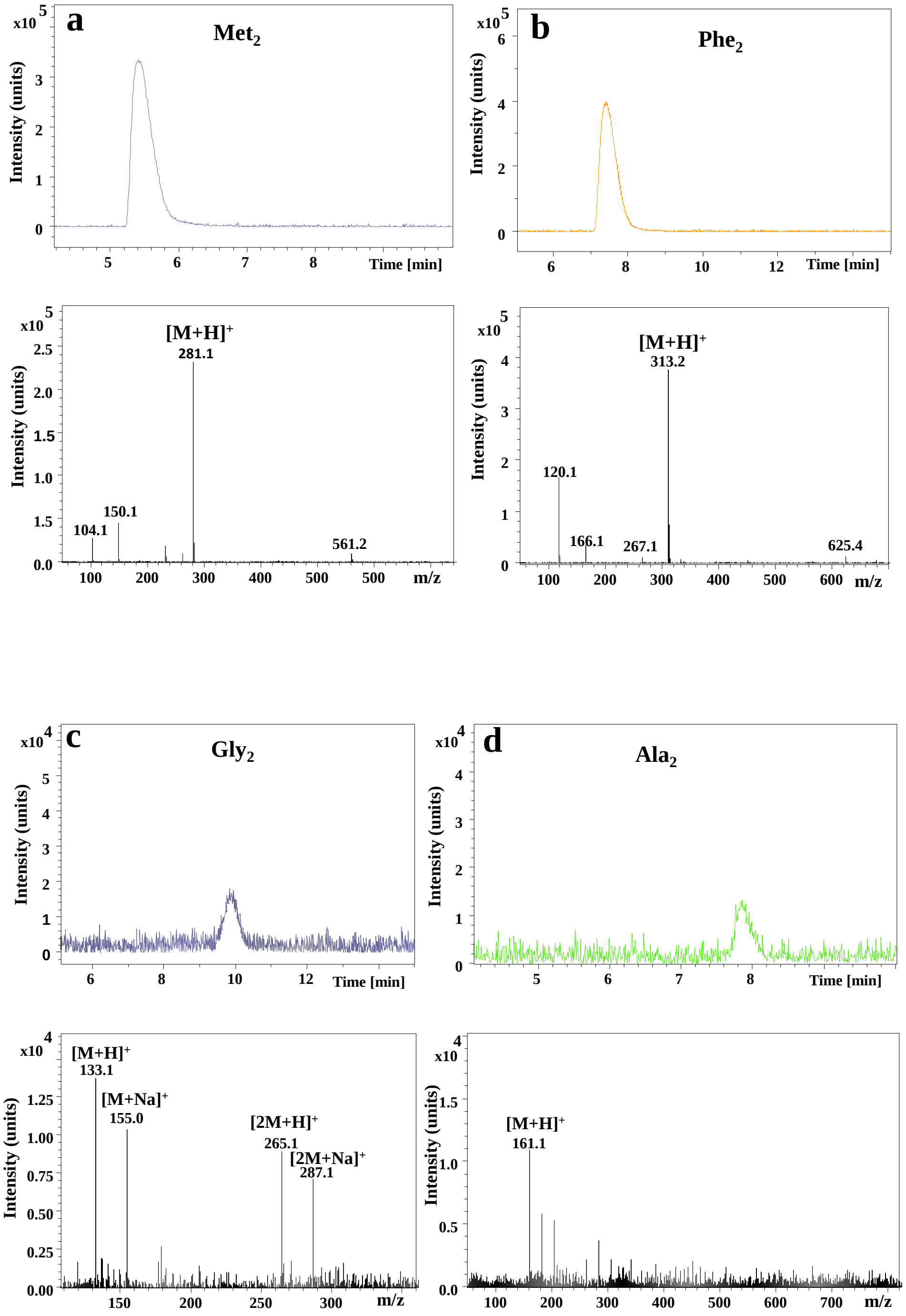

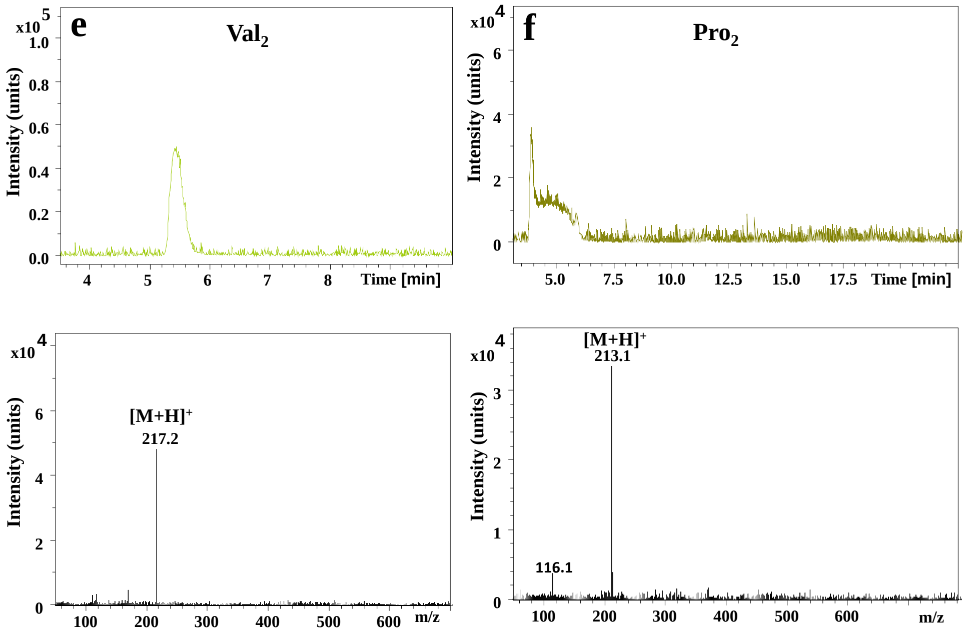

Fig. S1 HPLC-MS extracted ion chromatogram of the linear dipeptides $[M+H]^+$ ionic species for the reaction products of the amino acids with P_3m

The reaction of Met, Phe, Gly, Ala, Val, and Pro with P_3m gave the resulting dipeptides, but the reaction of Ser, Arg, His and Asp with P_3m showed no peptide formation. a) the reaction products of Met with P_3m; b) the reaction products of Phe with P_3m; c) the reaction products of Gly with P_3m; d) the reaction products of Ala with P_3m; e) the reaction products of Val with P_3m; f) the reaction products of Pro with P_3m.

3 NMR spectra of product cyclo-Pro-Pro

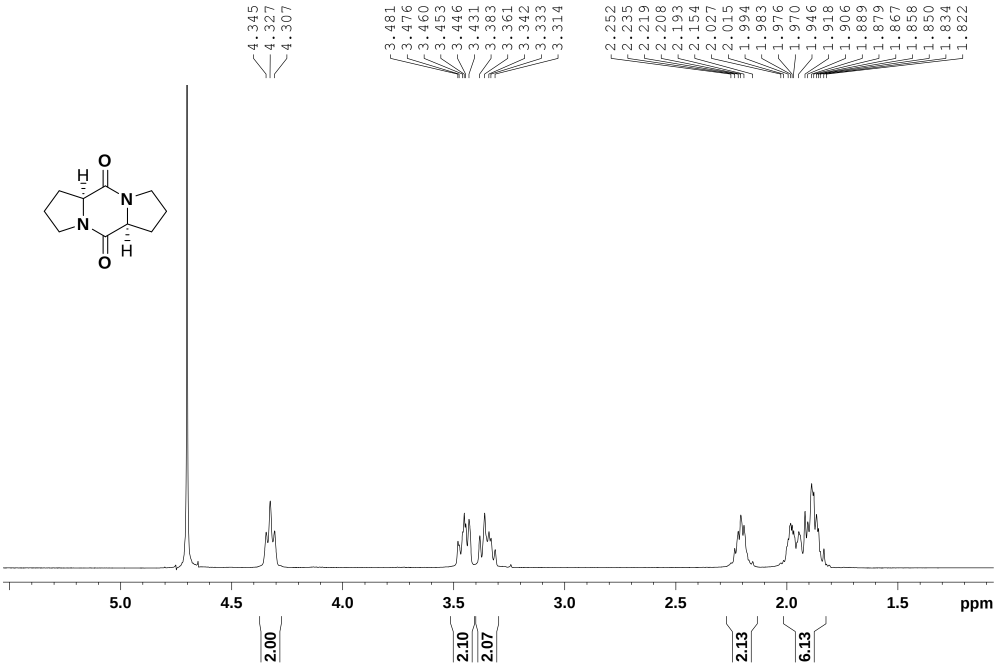

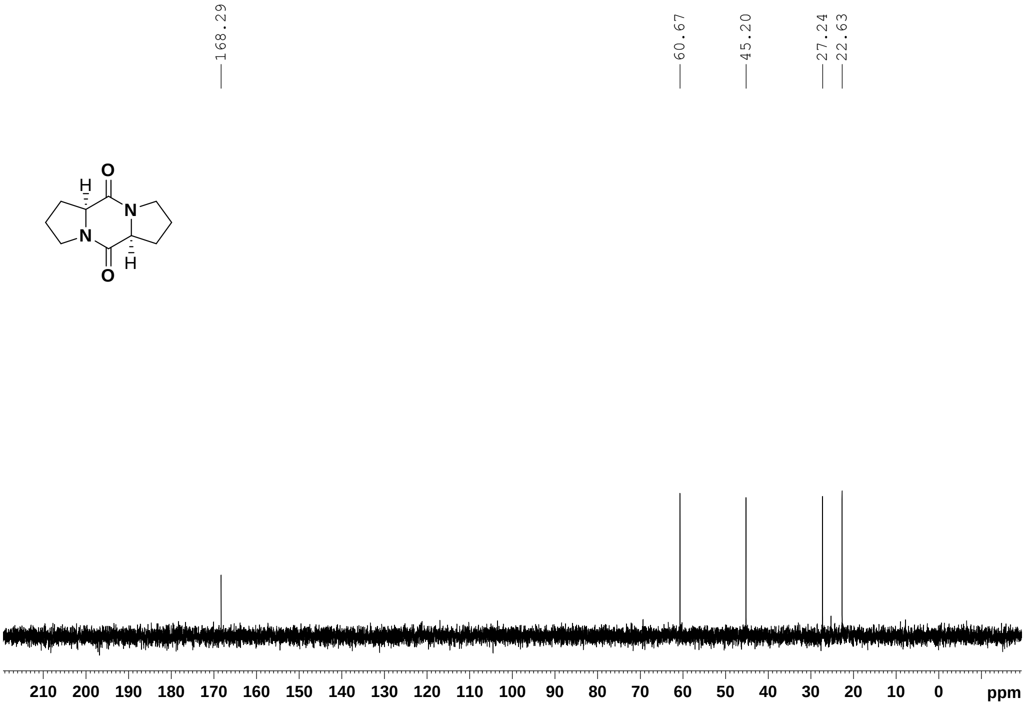

Fig. S2 NMR spectra for the reaction products cyclo-Pro-Pro

Octahydro-5H,10H-dipyrrolo[1,2-a:1',2'-d]pyrazine-5,10-dione. White solid; ^{1}H NMR (D_2O, 400 MHz): δ 4.32 (t, J = 7.6 Hz, 2H), 3.48-3.43 (m, 2H), 3.38-3.31 (m, 2H), 2.25-2.15 (m, 2H), 2.02-1.82 (m, 6H); ^{13}C NMR (D_2O, 100 MHz): δ 168.29, 60.67, 45.19, 27.23, 22.63.

4 Spectra of product cyclo-D-Pro-D-Pro

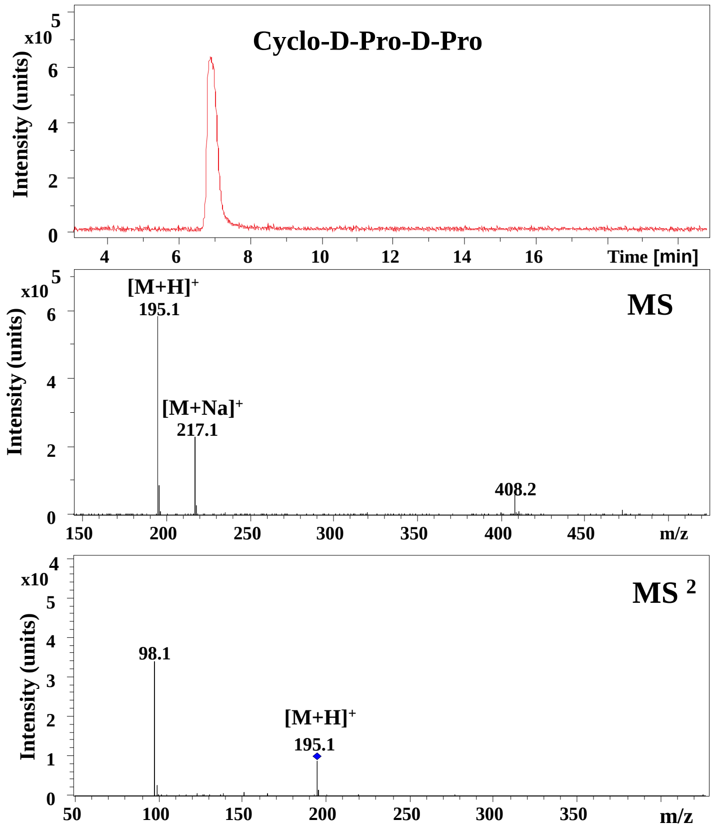

Fig. S3 HPLC-MS extracted ion chromatogram of the ion at *m/z* 195, corresponding to cyclo-D-Pro-D-Pro $[M+H]^+$ ionic species for the reaction products of D-Pro with P_3m

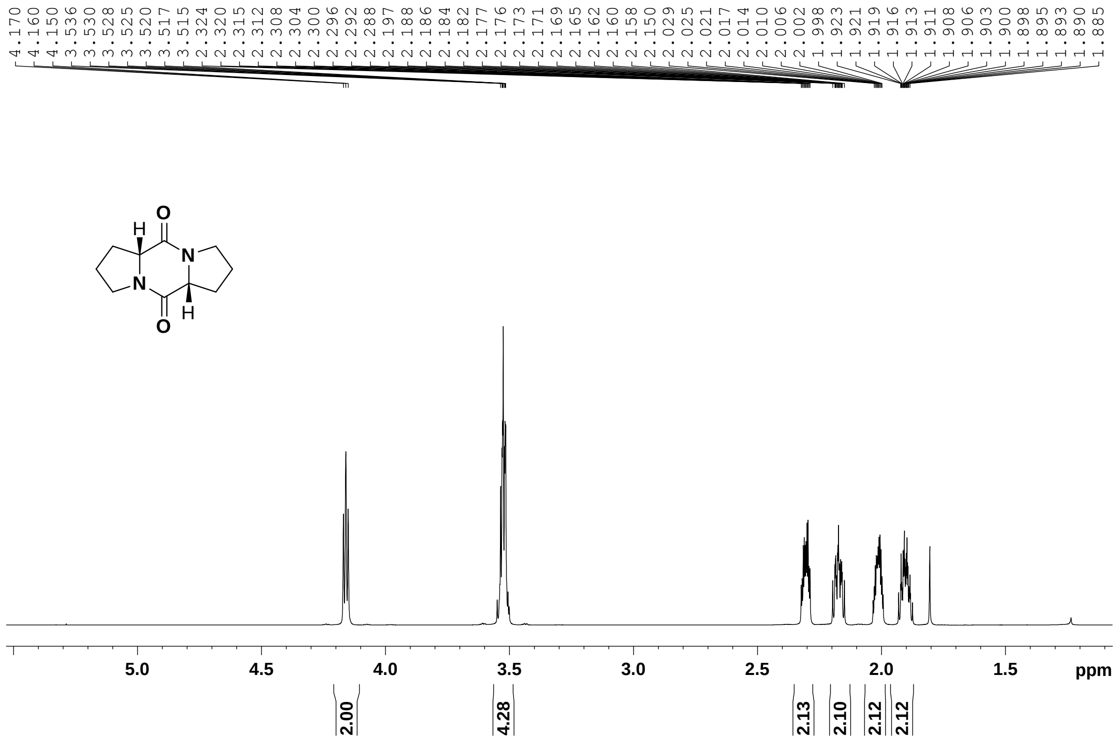

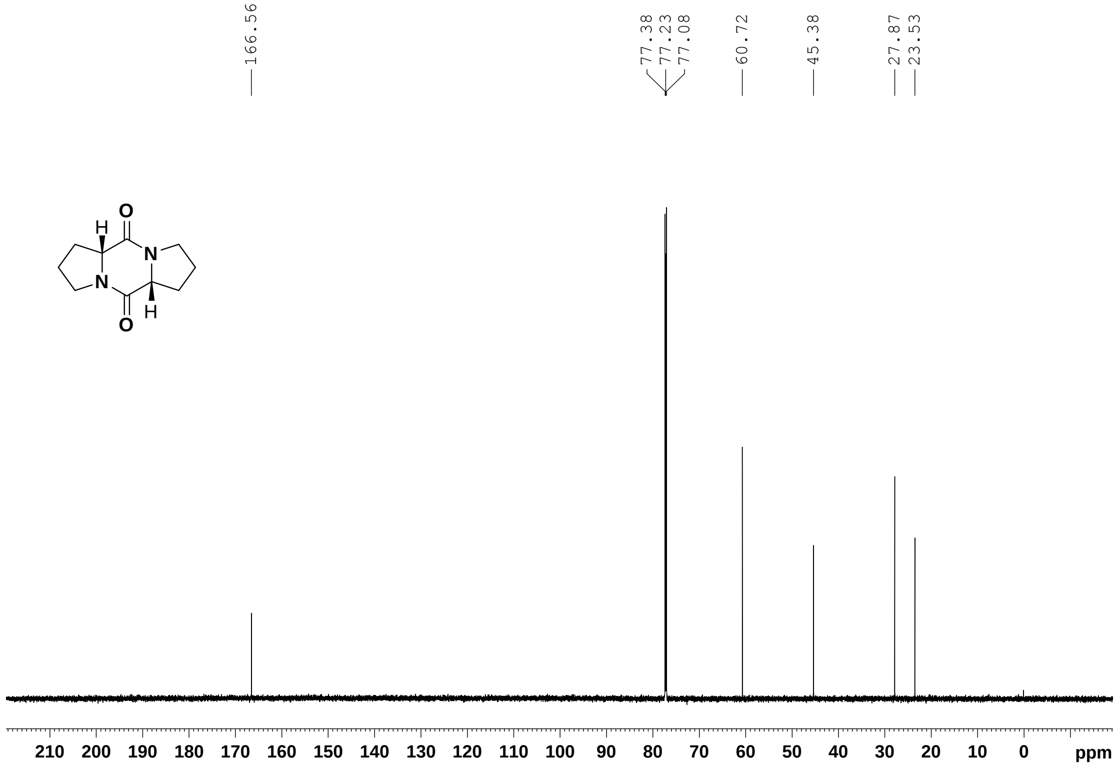

Fig. S4 NMR spectra for the reaction products cyclo-D-Pro-D-Pro

Octahydro-5H,10H-dipyrrolo[1,2-a:1',2'-d]pyrazine-5,10-dione. White solid; ^{1}H NMR ($CDCl_3$, 850 MHz): δ 4.16 (t, J = 8.21 Hz, 2H), 3.53-3.51 (m, 4H), 2.32-2.28 (m, 2H), 2.19-2.14 (m, 2H), 2.03-1.99 (m, 2H), 1.93-1.87 (m, 2H); ^{13}C NMR ($CDCl_3$, 212.5 MHz): δ 166.55, 60.71, 45.38, 27.87, 23.53.

5 Reaction products analysis of proline mixed with amino acids and P_3m

0.3 mmol sodium trimetaphosphate (P_3m) and 0.3 mmol proline were mixed with 0.3 mmol amino acids in 3 mL water, respectively. The pH of the reaction mixture was adjusted to 10~11 using 10 M NaOH. Then the reaction mixtures were placed at 35 ℃ for 7 d. The reactions were quenched by adding 6 M HCl. The crude products were filtered and analyzed by HPLC-MS/MS.

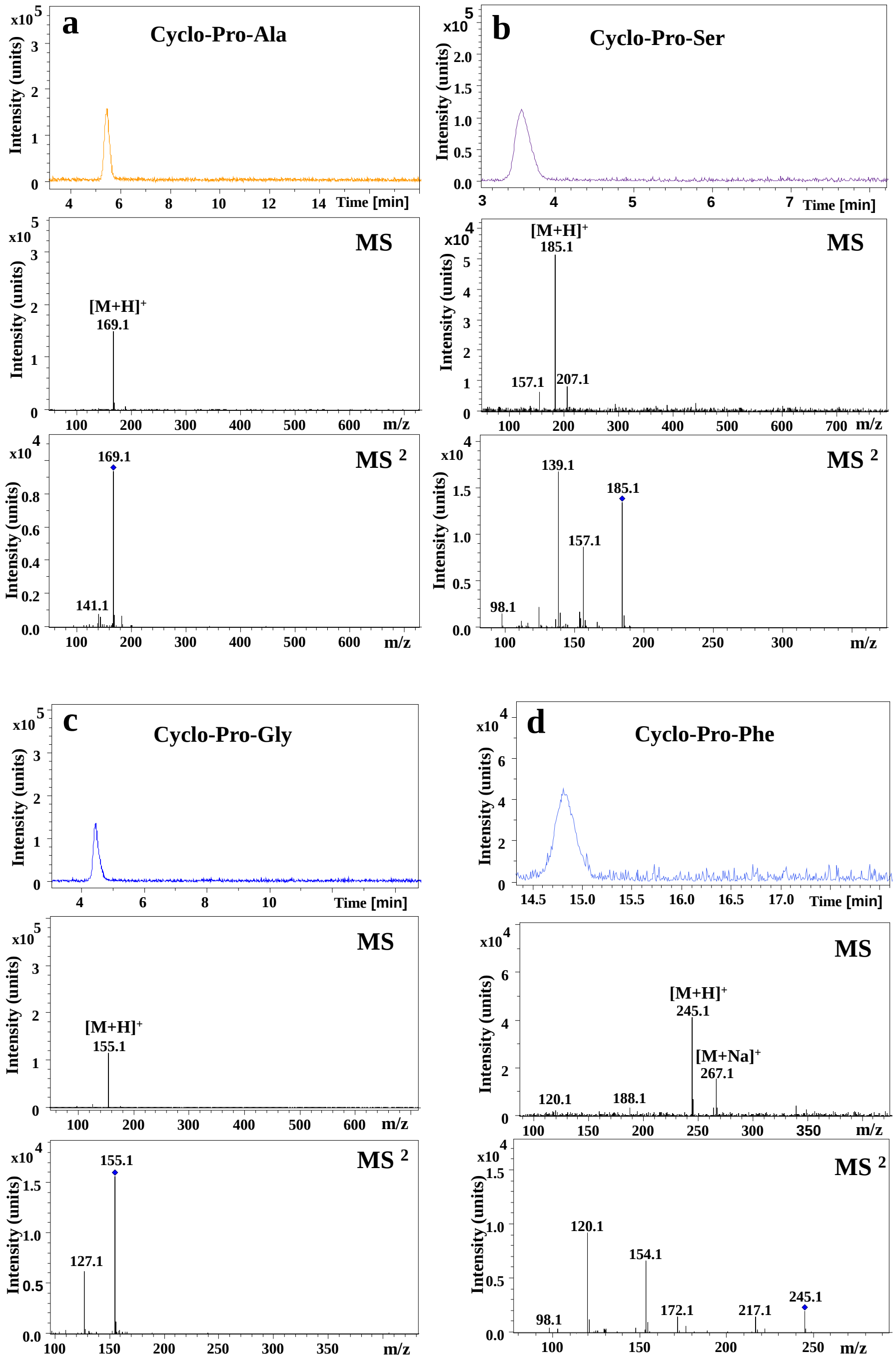

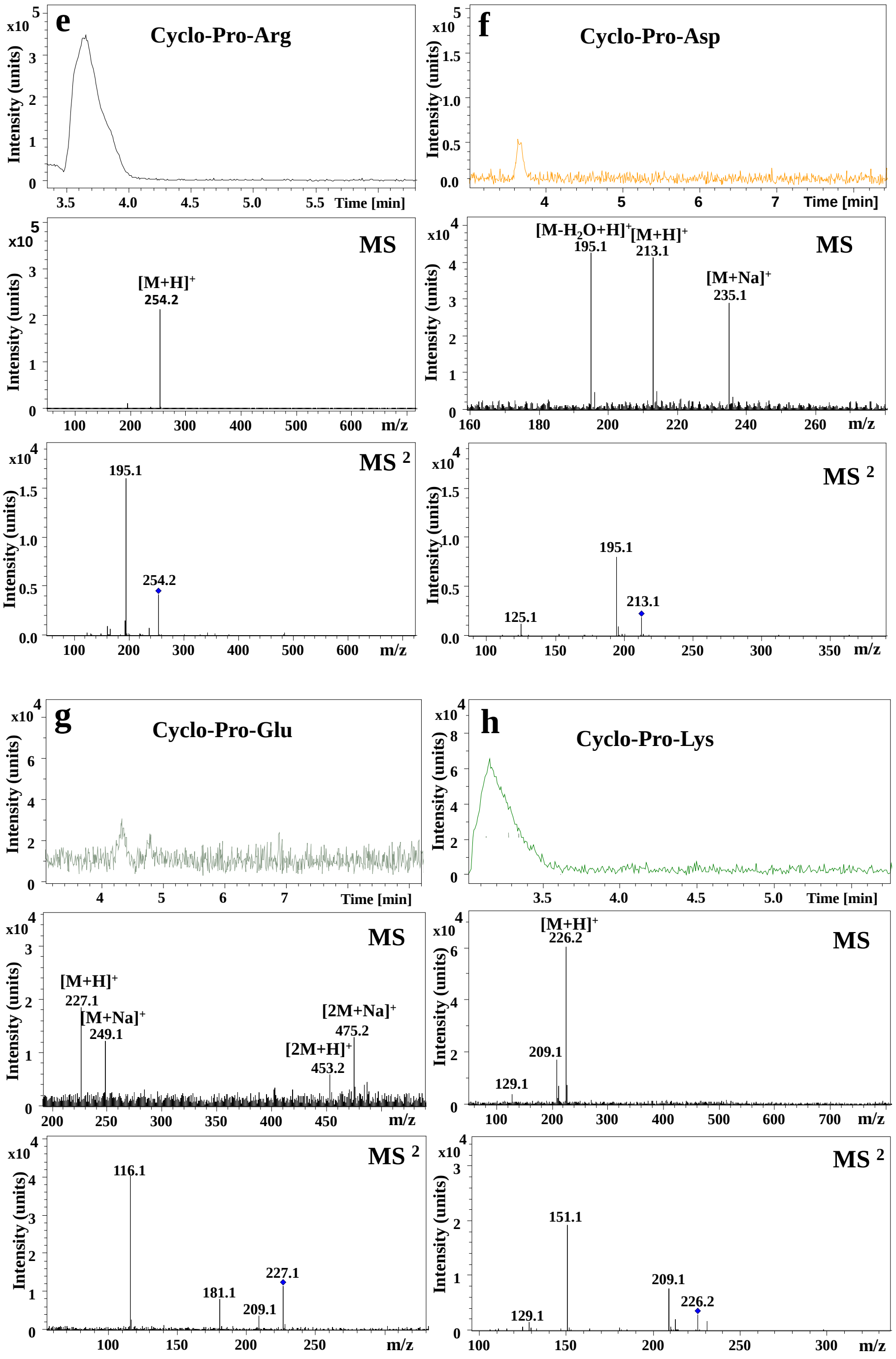
e
Cyclo-Pro-Arg
MS
[M+H]+
254.2
MS 2
195.1
254.2
f
Cyclo-Pro-Asp
MS
[M-H2O+H]+
195.1
[M+H]+
213.1
[M+Na]+
235.1
MS 2
125.1
195.1
213.1
g
Cyclo-Pro-Glu
MS
[M+H]+
227.1
[M+Na]+
249.1
[2M+Na]+
475.2
[2M+H]+
453.2
MS 2
116.1
181.1
209.1
227.1
h
Cyclo-Pro-Lys
MS
[M+H]+
226.2
209.1
129.1
MS 2
151.1
129.1
209.1
226.2
Intensity (units)
Time [min]
m/z

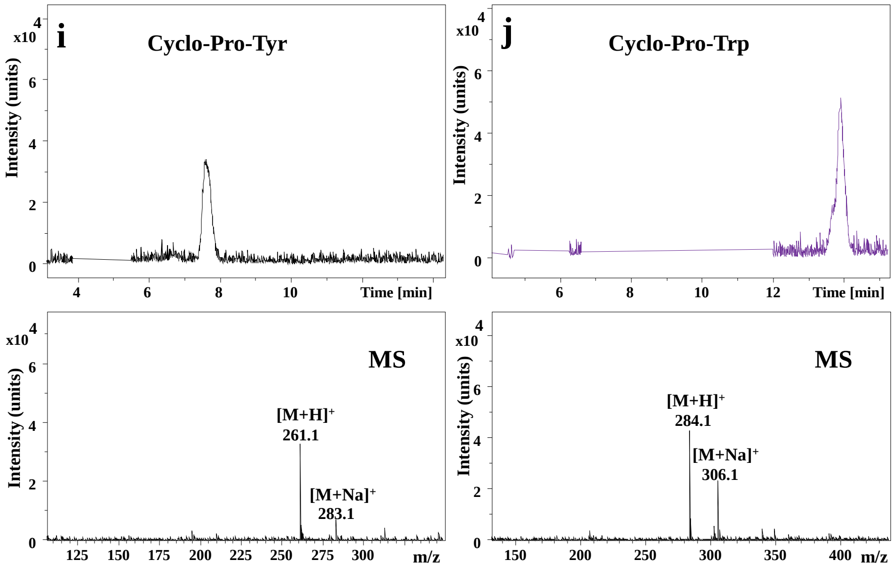

Fig. S5 HPLC-MS/MS extracted ion chromatogram of the cyclic dipeptides $[M+H]^+$ ionic species for the products of proline mixed with other amino acids and aqueous solution of P_3m.

a) the product cyclo-Pro-Ala of Ala with Pro at aqueous solution of P_3m; b) the product cyclo-Pro-Ser of Ser with Pro at aqueous solution of P_3m; c) the product cyclo-Pro-Gly of Gly with Pro at aqueous solution of P_3m; d) the product cyclo-Pro-Phe of Phe with Pro at aqueous solution of P_3m; e) the product cyclo-Pro-Arg of Arg with Pro at aqueous solution of P_3m; f) the product cyclo-Pro-Asp of Asp with Pro at aqueous solution of P_3m; g) the product cyclo-Pro-Glu of Glu with Pro at aqueous solution of P_3m; h) the product cyclo-Pro-Lys of Lys with Pro at aqueous solution of P_3m; i) the product cyclo-Pro-Tyr of Tyr with Pro at aqueous solution of P_3m; j) the product cyclo-Pro-Trp of Trp with Pro at aqueous solution of P_3m.

6 NMR spectra of product cyclo-Pro-Met

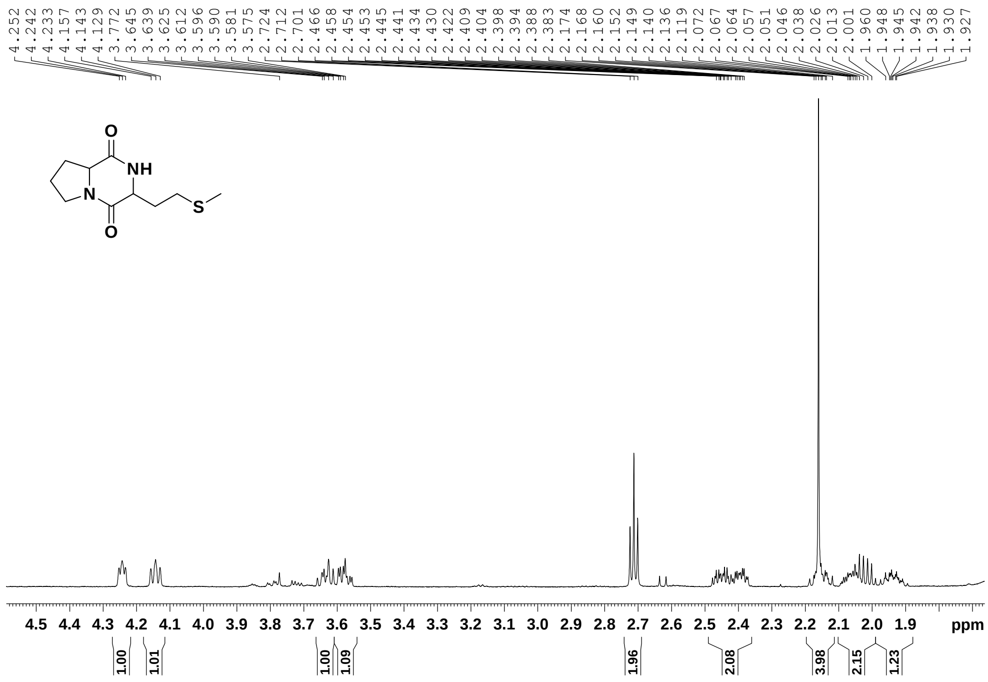

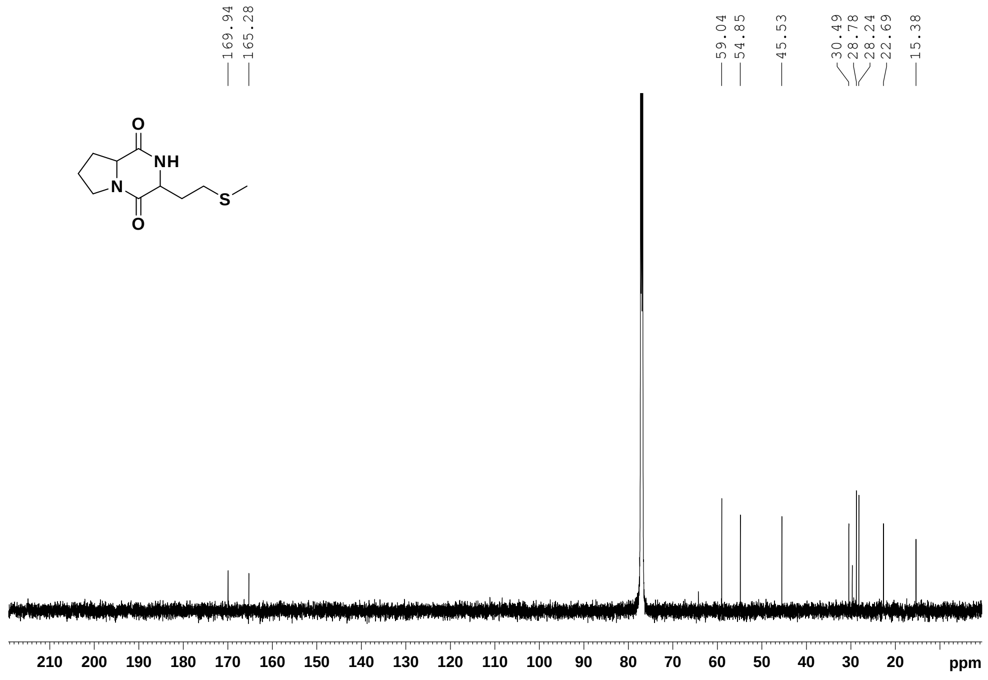

Fig. 6S NMR spectra for the reaction products cyclo-Pro-Met

3-(2-(methylthio)ethyl)hexahydropyrrolo[1,2-a]pyrazine-1,4-dione. White solid; ^{1}H NMR ($CDCl_3$, 600 MHz): δ 4.24 (t, J = 5.47, 1H), 4.15-4.12 (m, 1H), 3.65-3.61 (m, 1H), 3.59-3.55 (m, 1H), 2.71 (t, J = 6.84, 2H)2.47-2.37 (m, 2H), 2.18-2.12 (m, 4H), 2.09-1.97 (m, 2H), 1.96-1.89 (m, 1H); ^{13}C NMR ($CDCl_3$, 150 MHz): δ 169.9, 165.2, 59.0, 54.8, 45.5, 30.4, 28.7, 28.2, 22.6, 15.3.

7 NMR spectra of product cyclo-Pro-Gly

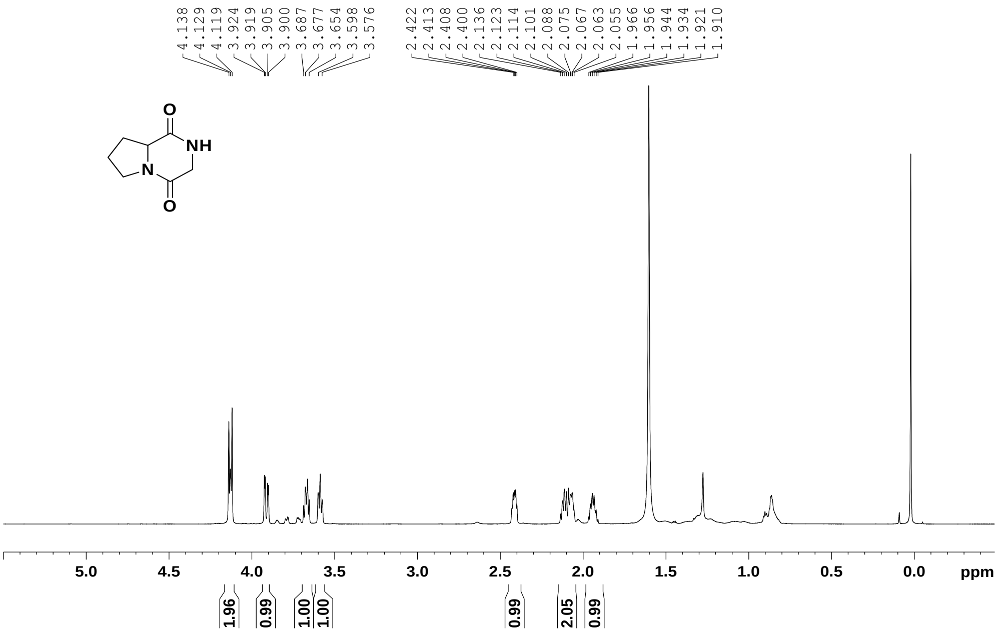

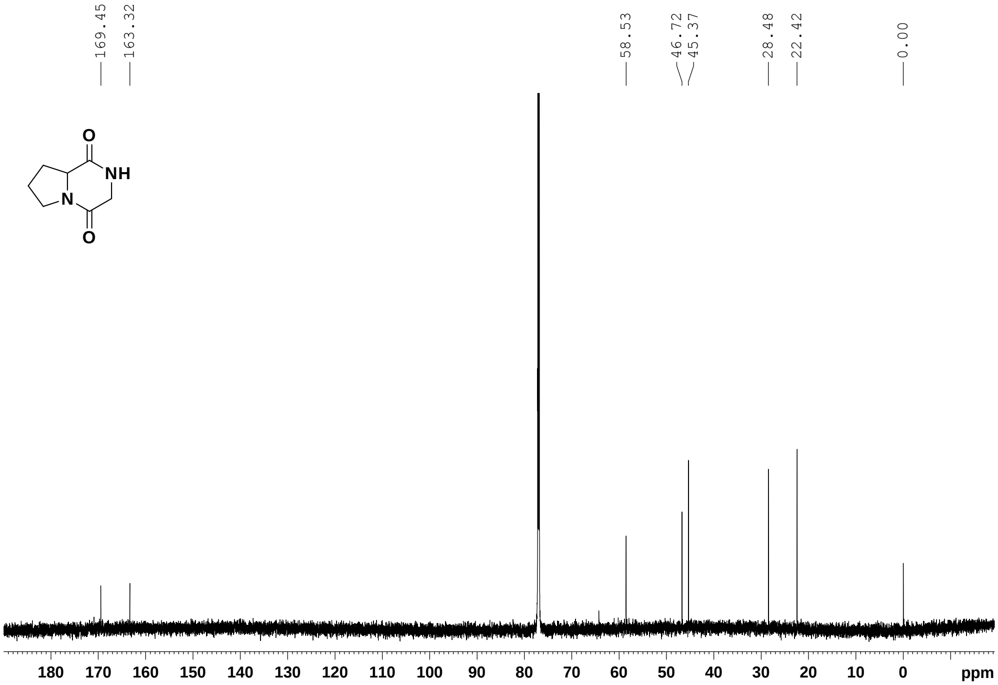

Fig. 7S NMR spectra for the reaction products cyclo-Pro-Gly

Hexahydropyrrolo[1,2-a]pyrazine-1,4-dione. White solid; ^{1}H NMR ($CDCl_3$, 850 MHz): δ 4.13-4.11 (m, 2H), 3.92-3.89 (m, 1H), 3.68-3.65 (m, 1H), 3.59-3.57 (m, 1H), 2.42-2.39 (m, 1H), 2.13-2.05 (m, 2H), 1.96-1.91 (m, 1H),; ^{13}C NMR ($CDCl_3$, 212.5 MHz): δ 169.4, 163.3, 58.5, 46.7, 45.3, 28.4, 22.4.

8 The crude products' NMR spectrum of hydroxyproline mixture with P_3m

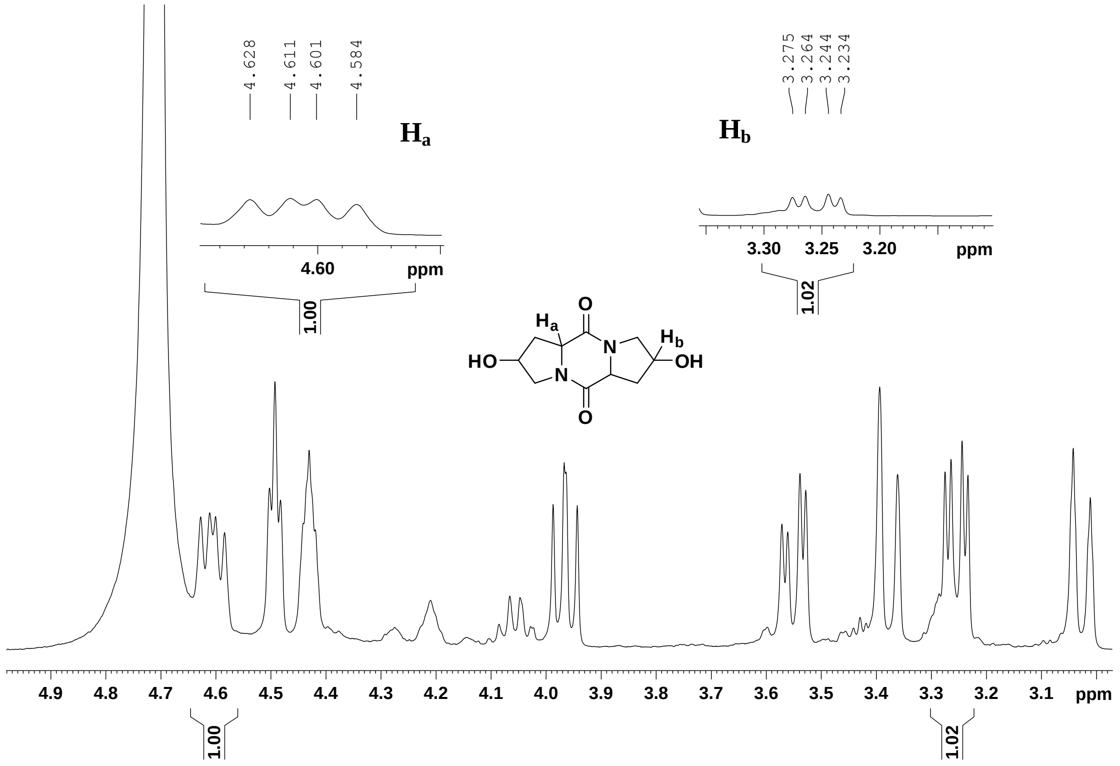

Fig. 8S NMR spectrum of the crude products of hydroxyproline mixture with P_3m
